# A recombinant rabies virus carrying GFP between N and P affects viral transcription in vitro

**DOI:** 10.1007/s11262-016-1313-2

**Published:** 2016-03-08

**Authors:** Jun Luo, Jing Zhao, Qin Tian, Weiyu Mo, Yifei Wang, Hao Chen, Xiaofeng Guo

**Affiliations:** College of Veterinary Medicine, South China Agricultural University, Guangzhou, China

**Keywords:** Rabies virus, Gene transcription, GFP, HEP-Flury, Vector

## Abstract

Several studies have demonstrated the rabies virus to be a perfect potential vaccine vector to insert foreign genes into the target genome. For this study, a green fluorescent protein (GFP) gene was cloned into the rabies virus (RABV) genome between the N and P gene. CT dinucleotide was inserted as intergenic region. The recombinant high egg passage Flury strain (HEP-Flury) of RABV, carrying GFP (rHEP-NP-GFP), was generated in BHK-21 cells using reverse genetics. According to the viral growth kinetics assay, the addition of GFP between N and P gene has little effect on the viral growth compared to the parental strain HEP-Flury. Quantitative real-time PCR (qPCR) indicated that rHEP-NP-GFP showed different viral gene transcription, especially for G gene, compared to HEP-Flury. The same is true for one other recombinant RABV carrying GFP between G and L gene in NA cells. In addition, parent HEP-Flury showed more expression of innate immune-related molecules in NA cells. Compared to HEP-Flury, Western blotting (WB) indicated that insertion of a foreign gene following N gene enhanced the expression of M and G proteins. According to the qPCR and WB, GFP expression levels of rHEP-NP-GFP were significantly higher than rHEP-GFP. This study indicates HEP-Flury as valid vector to express exogenous genes between N and P.

## Introduction

Rabies virus (RABV) causes a fatal neurological disease both in humans and animals and more than 55,000 humans die of rabies each year with most cases occurring in developing countries [1–3]. RABV, an unsegmented, negative-stranded RNA virus, belongs to the genus *Lyssavirus* of the family *Rhabdoviridae*. The RABV genome is approximately 12 kb in size and comprises five genes that are encoding nucleoprotein (N), phosphoprotein (P), matrixprotein (M), glycoprotein (G) and the RNA-dependent RNA polymerase (L) [4]. Ribonucleoprotein, formed by RNA and N, together with P and L forms the viral replication complex [5]. RABV G and M regulate the RNA synthesis and affect the RV pathogenesis by regulating virus replication and by facilitating cell-to-cell spread [6, 7]. M bridges the RNP and the cytoplasmic domain of G to form the bullet-shaped virion [8]. A leader sequence at the 3′ end and a trailer sequence at the 5′ end of the RABV genome are conservative. From the 3′ end, a gradient of transcription occurs that follows the gene order 3′-lerder-N-P-M-G-L-trailer-5′. This gradient results in the most abundant viral transcripts of N mRNAs and the least abundant transcripts of L mRNAs in infected cells [9, 10]. Sequences were not transcribed in the intergenic region (IGR), which in turn affects the transcription of the virus gene [11]. Different IGRs, including the amount and type of nucleotide, resulted in a transcription change of downstream and upstream genes in VSV [12]. The five genes of RABV are separated by four IGRs comprising different numbers of nucleotides (2nt, 5nt, 5nt, and 423nt). CT dinucleotide of RABV N/P profoundly improved the transcription of downstream genes without affecting the upstream gene compared to other intergenic regions (P/M, M/G, and G/L). For this study, CT dinucleotide was chosen as the intergenic region between N and the exogenous gene.

High flexibility and straightforward manipulation make RABV a desired vector. Both foreign proteins and additional glycoprotein have been expressed by recombinant RABVs that carry exogenous genes between the G and L genes [13–18]. Most of the studies indicated that viral growth was not affected by the insertion of exogenous genes between G and L while [17, 19]. Some either positively or negatively influenced the viral growth in vitro, which may be related to the specifications of foreign protein [17, 19]. The SRV9 strain of RABV was used as a vector for expression of exogenous genes between P and M, which lead to no influence on virus packaging and growth characteristics [20]. In addition, human immunodeficiency virus type 1 Gag (HIV-1 Gag) was expressed in RABV between N and P and showed no differences in virus titers compared to parent RABV. However, Western blotting revealed that the expression of HIV-1 Gag between N and P was not enhanced in comparison to the expression between G and L, although it was located further upstream [21].

The labeling protein green fluorescent protein (GFP) is directly visible in fluorescent microscopy. Recombinant RABV expressing GFP between G and L has been used for research on RV mechanisms and to test virus-neutralizing antibodies [22, 23]. In addition, the expression of foreign proteins generally affects the characteristics of recombinant RABV including pathogenicity, growth, immunogenicity, and expression of structural proteins. For this study, a GFP gene carrying the transcription initiation signal and the transcription termination polyadenylation signal was first cloned into the HEP-Flury genome at the site between N and P genes. The intergenic region of the CT dinucleotide was introduced between N and GFP. The expression levels of viral genes and GFP were explored in NA cells infected by recombinant RABVs or parent HEP-Flury. The data indicates that foreign protein can be expressed in abundance through genes inserted between N and P. Through this technique, the growth of RABV is not affected.

## Materials and methods

### Cells, viruses, and antibodies

Baby hamster kidney (BHK-21) cells were maintained in Dulbecco’s modified Eagle’s medium (DMEM) (Gibco, Invitrogen, China) and contained 10 % fetal bovine serum (FBS) (Gibco, Australia). Mouse neuroblastoma (NA) cells were cultured in RPMI 1640 medium (Gibco, Invitrogen, China) supplemented with 10 % FBS. HEP-Flury and rHEP-GFP were propagated in BHK-21 cells. rHEP-GFP, a recombinant virus that carries GFP between the G and L genes, was previously rescued from the constructed plasmid [24]. Antibodies against both the RABV N, RABV M, and RABV G proteins that were used for Western blotting were stored in our laboratory. Fluorescein isothiocyanate (FITC)-conjugate antibodies against the RABV N protein were purchased from Fujirabio Inc. (Malvern, PA). Antibodies against GFP were purchased from Beijing Emarbio Science & Technology Co., Ltd.

### Construction of full-length cDNA clones

**Fig. 1:**
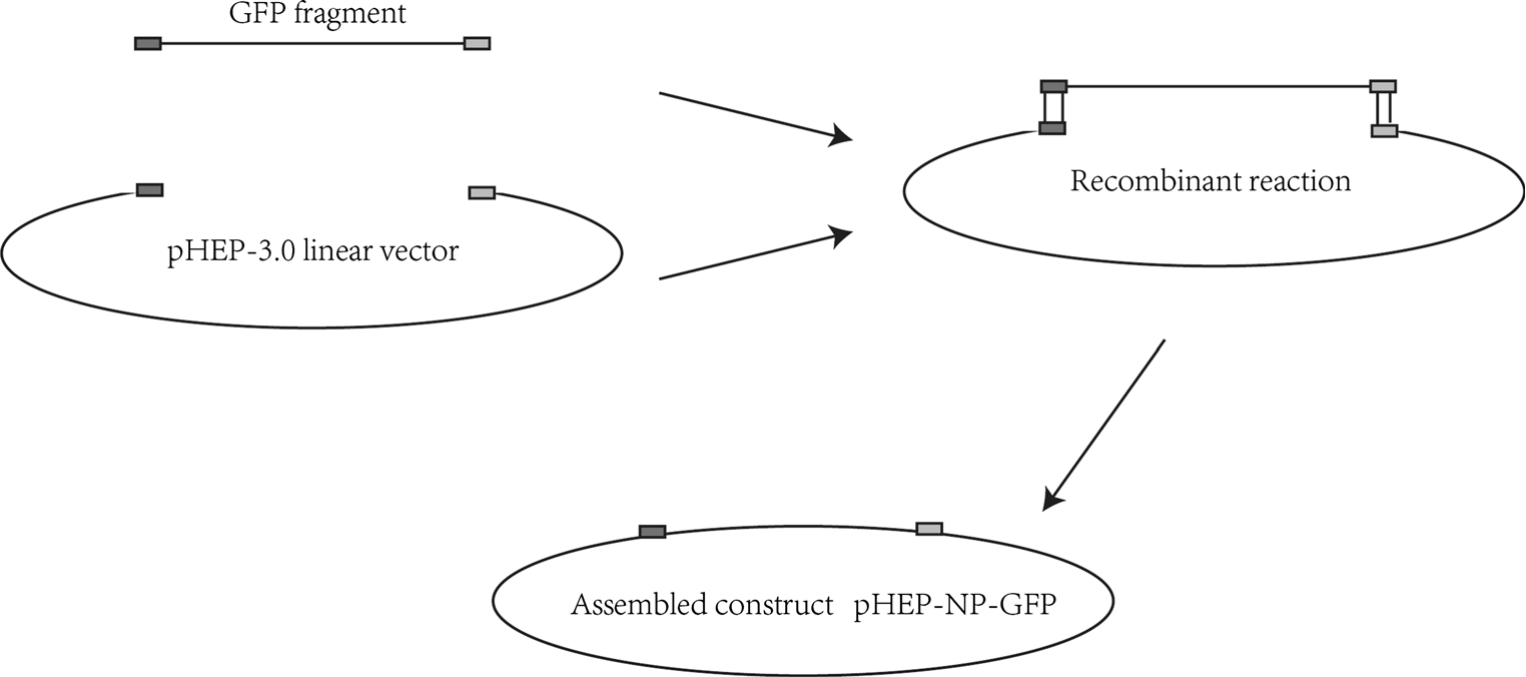
The linear vector was amplified using primers on the N and P genes with the template of plasmid pHEP-3.0 by PCR. GFP fragments were amplified carrying homolog’s sequences and reacted with the linear vector according to the manufacturer’s instructions

The recombinant RABV infectious clones were constructed by inserting the GFP gene between N and P genes. The fragment of GFP cDNA including transcription initiation signal (TIS) and transcription termination polyadenylation signal (TTP) was amplified by PCR using the primers: cGFP-forward (5′-ATCATGAAAAAAACTAACACCTCTCGTACGATGGCTAGCAAAGG-3′, homologous sequence underlined) and cGFP-reverse (5′-CGAAAGGAGGAGTGTTAGTTTTTTTCTCGACTGAAATGCT-3′, homologous sequence underlined). The linear vector was amplified from the rabies virus full-length cDNA vector (pHEP-3.0) using the primers: Li-NP-forward (5′-CTAACACTCCTCCTTTCGAACCATCCCAAGTATGAGCA-3′, homologous sequence underlined) and Li-NP-reverse (5′-AGTTTTTTTCATGATGGATATACACAATC-3′, homologous sequence underlined). The linear vector was used for the construction of recombinant RV cDNA clones. The GFP fragment was cloned into the corresponding site of the linear vector by a recombinant reaction using the ClonExpressMultiS One Step Cloning Kit (Vazyme Biotech CO., Ltd., Nanjing), following to the manufacture’s instructions. The route of cloning is shown in Fig. 1. Restriction analysis and DNA sequencing (Genwiz, Shuzhou) were used to identify the resulting plasmid (prHEP-NP-GFP).

### Rescue of recombinant virus from cDNA

Recombinant rabies virus was rescued as previously described [21]. Briefly, BHK-21 cells grown in 12-well plates (Corning, USA) were transfected with 1.0 µg of pHEP-NP-GFP plasmid, 0.25 µg of pH-N, 0.125 µg of pH-P, 0.05 µg of pH-L, and 0.075 µg of pH-G, respectively. The SuperFect transfection reagent (Qiagen, GER) was used according to the manufacturer’s protocol. After incubation at 37 °C for 4 days, the culture medium was removed and fresh medium was added to the cells. Incubation at 37 °C was kept until the green fluorescent spots became visible in fluorescence microscopy (after ~4 days). By this point, the cell culture medium was collected and viruses were detected using FITC-conjugated antibodies against the RABV N protein. Briefly, the cell culture medium was then discarded, and the cells were fixed with 80 % pre-cold acetone for 30 min at −20 °C. The cells were then washed three times with PBS and stained with FITC-conjugated anti-RABV N antibody at 37 °C. Foci were tested under a fluorescence microscope (AMG, USA). To confirm whether the rescued rRABV was derived from the cloned pHEP-NP-GFP, RT-PCR was performed using the primers: N-forward (5′-TGACAGGGTGCCAGAAAT-3′) and P-reverse (5′-TAGGAAAGTTGACCGAGA-3′).

### Virus titration

rRABVs (rHEP-GFP and rHEP-NP-GFP) were propagated in BHK-21 cells. For virus titration, monolayers of BHK-21 cells were infected in 96-well plates, using serial 10-fold dilutions of virus and incubated at 37 °C. 96-h post-infection, foci of GFP were counted under a fluorescence microscope and calculated as focus forming units/ml (FFU/ml).

### Virus multi-step growth assays

For the multi-step growth curve, monolayers of NA or BHK-21 cells were cultured in 9-cm cell culture dishes and were infected with various viruses at a multiplicity of infection (MOI) of 0.01 FFU. After 1 h of incubation at 37 °C, the inoculum was discarded and the cells were washed three times with phosphate-buffered saline (PBS). 8 mL fresh growth medium was added into cells followed by further incubation at 37 °C. Samples of culture supernatant were collected at 24, 48, 72, 96, and 120 h after infection. Virus titer was determined in BHK-21 cells as previously described.

### Quantitative real-time PCR

To investigate the expression of N, P, M, G, L, and GFP genes as well as that of innate immune-related molecules, BHK-21 cells were infected with various viruses at an MOI of 0.1. This was done in 6-well cell culture plate and incubation at 37 °C followed the infection. Cells were harvested and total RNA was extracted using the Total RNA Kit II (OMEGA, USA) according to the manufacturer’s protocol. Reverse transcription (RT) was carried out using the Transcriptor First Strand cDNA Synthesis Kit (Roche, GER) according to the manufacturer’s instructions. Each reaction was carried out in triplicate using SYBR Green Master Mix (Vazyme Biotech CO., ltd., Nanjing) and following the manufacturer’s instructions. Quantitative real-time PCR (qPCR) was carried out in a CFX384 Real-Time System (Bio-Rad, USA). To analyze the expression of target genes and virus genome, Glyceraldehyde-3-phosphate dehydrogenase (GAPDH) was used as reference gene. The primers used to amplify target and reference genes are shown in Table 1.

**Table 1:** Primers used for the analysis of genes expression [17]

Gene	Left primer (5′-3′)	Right primer (5′-3′)
RABV N	TTTAGTCGGTCTTCTCCTGAGTCT	AATCTGCTCTATTCTATCCGCAATGT
RABV P	GAGTCCAAATAGTCAGACAAATGAGGT	AGGAAAGTTGACCGAGACATAGGA
RABV M	AGAGGACAAAGACTCTTCTCTGCT	TGGAGTTAAGCCCGTATGTTCTCT
RABV G	GCCTTGATTGCCCTGATGTTGATAA	CATTTCTCCCTGTCCCTCCAAGAT
RABV L	TGTTGATGTCTGATTTCGCATTGTCT	AAGGGAACGCTCTTGACAGATGT
RABV Genome	AGAAGAAGCAGACATCGTCAGTTG	GGAGACCACCTGATTATTGACTTTGA
GFP	TGATGTTAACGGCCACAAGTTCTC	CAGTTTGCCAGTAGTGCAGATGAA
ISG20	TACTACAGCCGAGTGTCCCTG	GGCATCTTCCACAGAGCAGT
STAT1	TCCCGTACAGATGTCCATGAT	CTGAATATTTCCCTCCTGGG
IFN a1	GAGAAGAAACACAGCCCCTG	TCAGTCTTCCCAGCACATTG

### Western blot

NA cells cultured in 6-well plates were infected with different viruses at an MOI of 0.1. After incubation at 37 °C for 24 and 72 h, protein samples were harvested to investigate the expression of proteins RAV N, RABV M, RABV G, and GFP in vitro. Briefly, NA cells were washed three times with PBS after the discarding of culture medium and then lysed on ice using RIPA buffer (Beyotian, China). The lysates were centrifuged at 13,000 rpm for 20 min and all supernatants were collected. Proteins were separated by SDS-PAGE, and subsequently transferred on to PVDF membranes and incubated with antibodies against RABV N, RABV M, RABV G, GFP, or β-actin. After a second incubation with goat anti-mouse antibody labeled with horseradish peroxidase (HRP), the membranes were stained with BeyoECL Plus A and B (Beyotime, China) according to the manufacturer’s instructions. Protein fingerprints were shown using Fine-do ×6 (Tanon, China).

## Results

### Generation of recombinant RABV expressing GFP between N and P genes

**Fig. 2:**
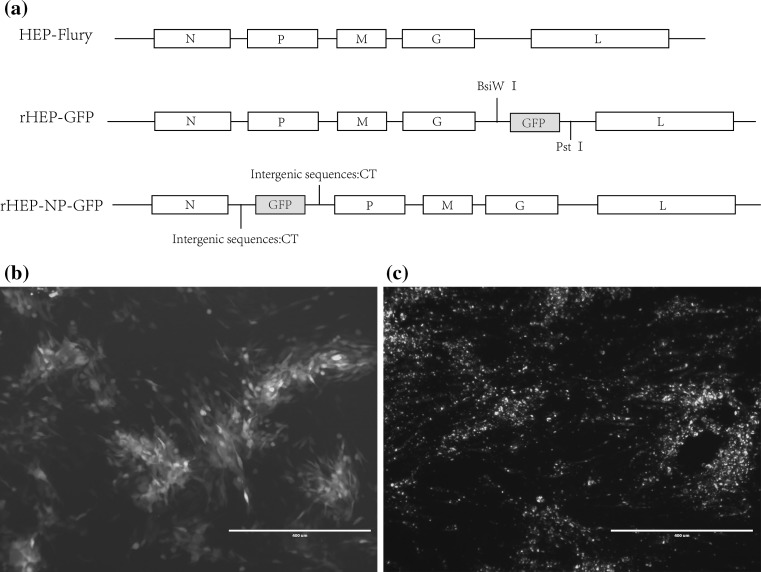
Generation of recombinant RABV expressing GFP between N and P genes. **a** The recombinant RABV (rRABV) expressing GFP between N and P was constructed based on the HEP-Flury (see Sect. 2.2). The rRABV contains an additional transcription initiation/termination polyadenylation signal and CT dinucleotide intergenic region between the N and P genes using seamless cloning. **b** Test of expression of rHEP-NP-GFP. BHK-21 cells were infected with rRABV at an MOI of 0.1. GFP expression was identified at 48 h post-infection directly under a fluorescence microscope without antibody. **c** rRABV was confirmed. FITC-conjugated anti-RABV N antibody was used for the test of rRABV on BHK-21 cells post-infection

Previous studies have shown that intergenic regions play an important role in gene expression [25]. The GFP gene with a TIS and a TTP was cloned into the pHEP-3.0 genome (see Fig. 2a). For this study, CT dinucleotide was inserted as intergenic region between N and GFP, as well as between GFP and P. Sequencing the fragments within the infectious clones confirmed whether the insertion of the GFP was successful. The recombinant RABV was collected from BHK-21 cells using previously described procedures [26], resulting in rHEP-NP-GFP. A second recombinant RABV carrying the GFP gene between G and L was previously rescued by Liu Xiaohui et al. [24] and was named rHEP-GFP. This GFP had the same TIS and TTP as rHEP-NP-GFP. The GFP expression of rHEP-NP-GFP was directly measured (Fig. 2b) using BHK-21 cells at 48 h post-infection by means of fluorescence microscopy. To determine the rescued rRABV content, direct fluorescent antibody assay (dFA) was conducted with FITC-conjugated anti-RABV N antibody on BHK-21 cells (Fig. 2c).

### Virus multi-step growth assays

**Fig. 3:**
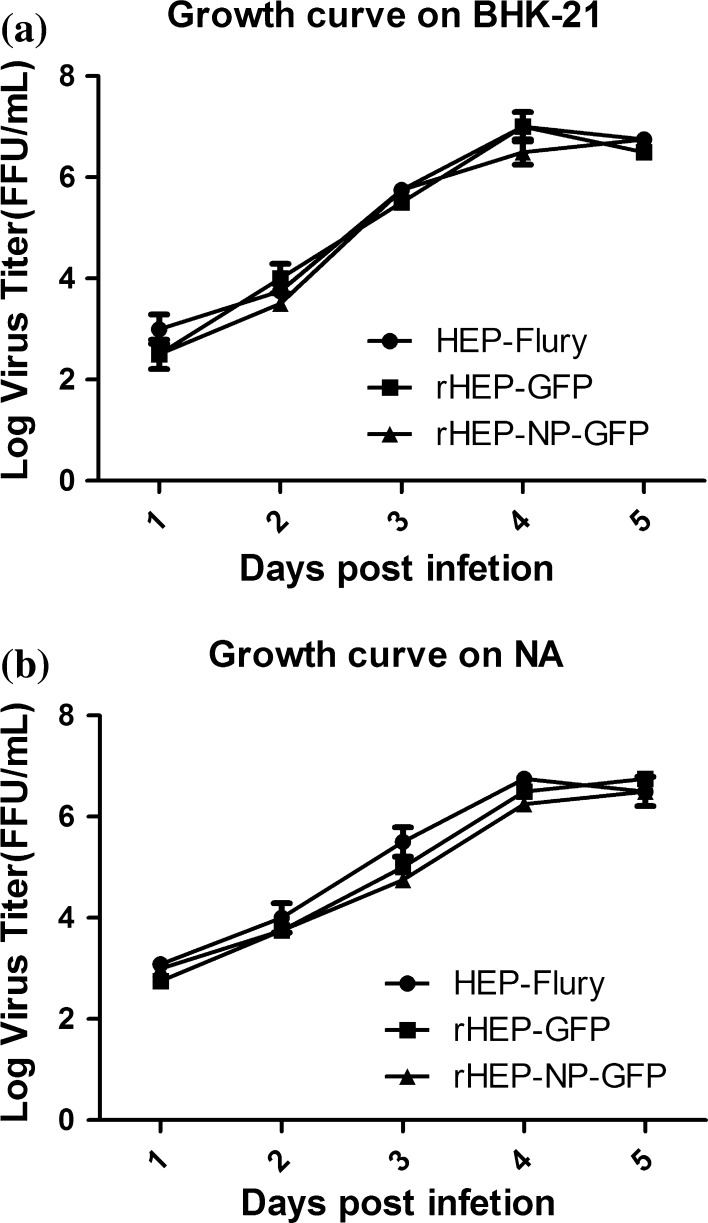
Growth curves of rRABVs (rHEP-GFP and rHEP-NP-GFP) and parental virus (HEP-Flury) in BHK-21 (**a**) and NA (**b**) cells. BHK-21 cells or NA cells were infected with HEP-Flury, rHEP-GFP, or rHEP-NP-GFP, respectively, at a multiplicity of infection (MOI) of 0.01. At days 1, 2, 3, 4, and 5 after infection, culture supernatants were harvested and virus titers were determined

To determine the growth characterization of rHEP-NP-GFP, viral kinetics were examined in both BHK-21 cells and NA cells. As shown in Fig. 3, no difference between the values was detected except for rHEP-NP-GFP reaching the highest titer at day 5. This study indicates that the late rHEP-NP-GFP titers were affected by the insertion of the exogenous gene in both BHK-21 cells and NA cells. Recombinant rHEP-NP-GFP showed a slower virus production, which may be related to the changes in the ratio of RABV proteins.

### Quantitative RT-PCR analysis of viral transcription and induction of innate immune-related molecules in vitro

**Fig. 4:**
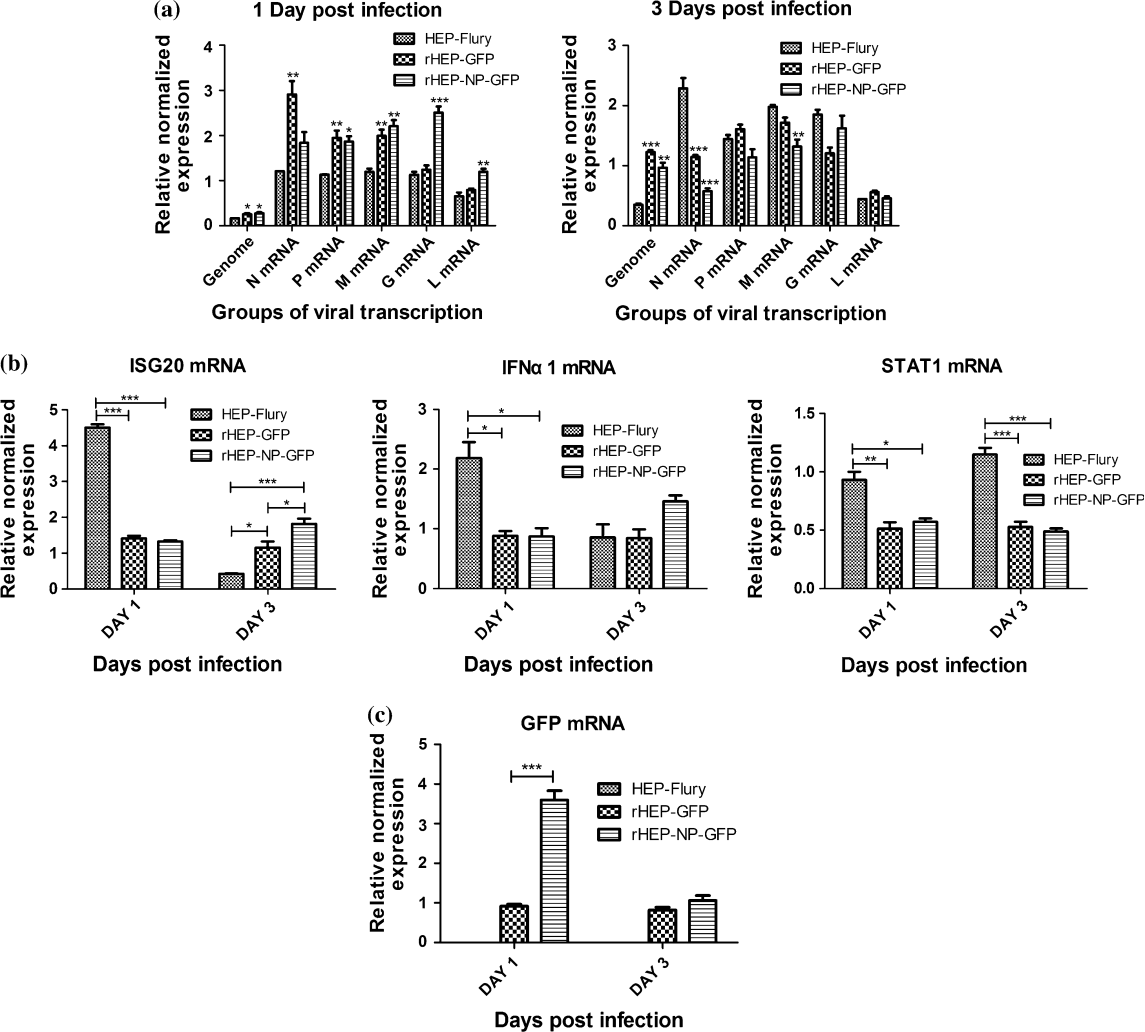
(**a**, **b**, **c**) Expression of viral genes, GFP, and innate immune-related molecules in vitro. NA cells were infected with HEP-Flury, rHEP-GFP, or rHEP-NP-GFP at an MOI of 0.1, and at 1 day post-infection (dpi) or 3 dpi. Cells were harvested for measuring the expression of viral genes (N, P, M, G, L, and genomic RNA), GFP, and innate immune-related molecules (ISG20, MusIFNα1, and STAT1) by means of quantitative real-time PCR. All expression of target genes was normalized to the expression level of the reference gene glyceraldehyde-3-phosphate dehydrogenase (GAPDH) by means of calculation of 2^−△△CT^. Data were analyzed using Bio-Rad CFX Manager and GraphPad Prism 5. Asterisks indicate significant differences of rHEP-GFP and rHEP-GFP compared to HEP-Flury (**a**) and differences among the experimental groups (**b**, **c**), as calculated by one-way ANOVA (*p < 0.05; **p < 0.01; ***p < 0.001)

In order to investigate the effect of both viral transcription as well as induction of innate immune-related molecules (caused by insertion of exogenous gene in vitro), NA cells were infected with HEP-Flury, rHEP-GFP, or rHEP-NP-GFP, respectively; at an MOI of 0.1 and at 1 dpi or 3 dpi, cells were harvested to determine the expression of viral genes (N, P, M, G, L, and genomic RNA), GFP, and innate immune-related molecules (ISG20, IFNα1, and STAT1). Quantitative real-time PCR was used for this as described previously [17]. As Fig. 4a shows, the expressions of all viral genes of HEP-Flury were less than those of rHEP-NP-GFP at 1 dpi while it increased at 3 dpi. Viral genome of HEP-Flury was expressed at a lower level at 3 dpi compared to rHEP-NP-GFP. Throughout all levels, rHEP-GFP showed irregular transcription compared to HEP-Flury and rHEP-NP-GFP. In addition, rHEP-NP-GFP showed a much higher level of G expression than HEP-Flury and rHEP-GFP at 1 dpi, although its G gene was distant from promoter. This may be due to the insertion of the CT dinucleotide intergenic region between N and GFP [12], which may indirectly affect the transcription of RABV G. Details about mRNA expression of innate immune-related molecules are shown in Fig. 4b. HEP-Flury showed a higher expression inducing the ability of ISG20, IFNα1, and STAT1 than rHEP-GFP and rHEP-NP-GFP at 1 dpi. At 3 dpi, NA cells infected by rHEP-NP-GFP expressed more ISG20 and IFNα1 than rHEP-GFP and HEP-Flury. The difference of GFP expression between rHEP-GFP and rHEP-NP-GFP is shown in Fig. 4c. At 1 dpi, GFP was profoundly more expressed (about 3 fold) in NA cells infected by rHEP-NP-GFP compared to rHEP-GFP.

### Western blot analysis of GFP, N, M, and G proteins expression in NA cells

**Fig. 5:**
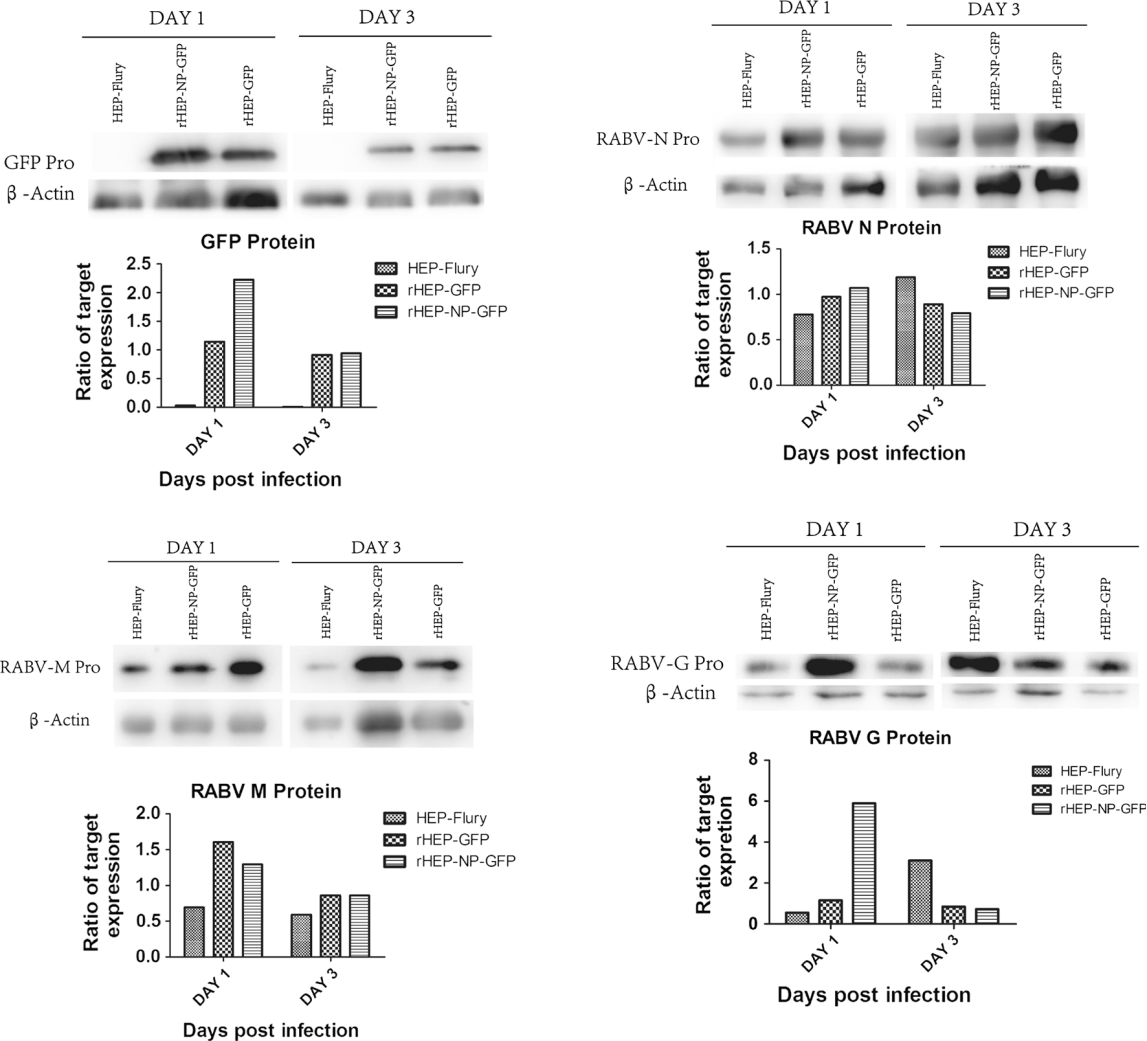
Western blot analysis of GFP, N, M, and G proteins expression in NA cells infected with RABVs. NA cells were infected with HEP-Flury, rHEP-GFP, or rHEP-NP-GFP at an MOI of 0.1, and at day 1 or day 3, total proteins of cells were harvested to investigate the GFP, N, M, and G protein expression using anti-GFP antibodies, anti-RABV N antibodies, anti-RABV M antibodies, or anti-RABV G antibodies, respectively. Gray analysis was conducted using Image J and the data for measuring the expression of target proteins was analyzed using GraphPad Prism 5

Expression of GFP, N, M, and G proteins was analyzed in NA cells infected with HEP-Flury, rHEP-GFP, and rHEP-NP-GFP. Western blotting was used for this analysis, with target protein expression relative to β-actin (Ratio of GFP/β-actin, N protein/β-actin, M protein/β-actin, and G protein/β-actin). Figure 5 shows the time-dependent GFP expression rates with significantly higher rates for rHEP-NP-GFP compared to rHEP-GFP for day 1. RABVs (HEP-Flury, rHEP-GFP, and rHEP-NP-GFP) showed a different ability in expression of N, M, and G protein. On day 1, N, M, and G proteins showed lower expression rates in HEP-Flury-infected NA cells compared to rRABVs. A comparison of expression rates of N and M proteins between rHEP-GFP and rHEP-NP-GFP showed no significant differences. The exception was M protein of rHEP-GFP, which is expressed at a higher level compared to that of rHEP-NP-GFP (at 1 dpi). The G protein expression of rHEP-NP-GFP was profoundly higher compared to HEP-Flury and rHEP-GFP at 1 dpi.

## Discussion

The intergenic region (IGR), which could not be transcribed, plays an important role in affecting downstream RNA transcription [12]. Recent research indicates regulation of L gene expression of RABV by the intergenic regions between G and L [25]. Five genes of RABV are separated by four IGRs comprising different numbers of nucleotides, 3′-5′ namely, 2, 5, 5, and 423 nucleotides. In this study, intergenic nucleotides (CT) were cloned into N and GFP in order to enhance GFP expression as well as other downstream genes.

Previous studies propose RABV to be a promising viral vector to express exogenous genes [27]. GFP, as a reporter gene, has been introduced into RABV between P and M or G and L [20, 22–24]. For this study, GFP was cloned into a HEP-Flury strain of RABV between N and P. Real-time PCR and Western blot were conducted to investigate the expression rates of rHEP-NP-GFP GFP in NA cells. As shown in Figs. 4 and 5, expressions of both GFP mRNA and protein of rHEP-NP-GFP were significantly higher compared to rHEP-GFP. This may be due to the location of GFP between N and P which is closer to the promoter region [28]. Other studies indicate that in VSV and RABV, both amount and type of nucleotides of intergenic sequences affect the transcription of downstream as well as upstream genes [12, 25]. CT dinucleotide as intergenic region increases the transcription of downstream genes.

In previous studies, the glycoprotein (G) of RABV was confirmed to play an important role in cytotoxicity and induction of virus-neutralizing antibodies (VNA) [13, 29]. Another study indicated that RABV G may not be crucial in pathogenicity and the expression of other viral proteins was at a higher level while the expression of G was reduced [30]. As shown in Figs. 4a and 5, the transcription and translation of rHEP-NP-GFP G gene is significantly higher than HEP-Flury and rHEP-GFP (at an early stage). This may be one cause for decreased rHEP-GFP N expression. The enhanced G protein expression of rHEP-NP-GFP may lead to more VNA in vivo [19], which could protect host from street strain of RABV. In addition, rHEP-NP-GFP showed an increased transcription of viral N, P, and M at 1 dpi compared to the parent strain HEP-Flury. This may be due to the increased activity of transcription caused by the insertion of CT dinucleotide between N and GFP. However, the RNA synthesis of RABV is regulated by viral proteins, which could not be produced without limit [6]. The decreased transcription of rHEP-NP-GFP at 3 dpi may correlate with the negative regulation of viral proteins.

In the study, ISG20 was expressed at a higher level in HEP-Flury at 1 dpi compared to rRABVs. ISG20 is a product stimulated by IFN and has been demonstrated to inhibit viral transcription and replication [31]. This may be another reason for the lower viral proteins expression and replication rates of HEP-Flury compared to rRABVs. In previous researches, RABV P was demonstrated to be an IFN antagonist that may decrease IFN gene expression and interferon-induced STAT signaling [32, 33]. Our study indicates that expressions of IFNα1 and STAT1 were higher in NA cells that have been infected with HEP-Flury compared to rHEP-GFP and rHEP-NP-GFP. This may be related to the higher expression of rHEP-GFP and rHEP-NP-GFP P.

It has been shown that N, P, and L were related to virus RNA replication and transcription [34, 35], while M plays an important role in virus assembling and budding [8]. This indicates that changes of the structural protein of RABV may affect the synthesis of RNA and virus replication. In this study, HEP-Flury has a stronger ability to express N and M compared to rHEP-NP-GFP (in the late stage). This may be the reason that leads to an earlier maximum in virus titer compared to rHEP-NP-GFP in NA cells.

In summary, to improve the expression of exogenous genes, CT dinucleotide was introduced as the intergenic region of N and GFP. This study shows that the rHEP-NP-GFP (carrying GFP gene between N and P) significantly affects the viral gene expression in vitro compared to parent HEP-Flury and rHEP-GFP. Viral growth was not affected by inserting GFP between N and P. Exogenous gene expression was higher between N and P than between G and L. This study indicates enhanced suitability of the N/P site for the expression of exogenous genes compared to the G/L. Moreover, it provides a foundation for gene research of the rabies virus.
